# Elucidating causal relationships of diet-derived circulating antioxidants and the risk of non-scarring alopecia: A Mendelian randomization study

**DOI:** 10.1097/MD.0000000000038426

**Published:** 2024-06-14

**Authors:** Yuchen Ba, Lele Shen, Xiangning Peng, Yujin Zhang, Junwen Wang

**Affiliations:** a The Second Hospital of Hunan University of Chinese Medicine, Changsha, Hunan, China; b The First Hospital of Hunan University of Chinese Medicine, Changsha, Hunan, China.

**Keywords:** derived antioxidants, diet, Mendelian randomization, non-scarring alopecia

## Abstract

Previous observational studies revealed controversy about the effect of circulating antioxidants on risk of alopecia. In the present study, we investigated the causal relationships between diet-derived circulating antioxidants and 2 non-scarring alopecia using Mendelian randomization (MR). Instrumental variables for antioxidants (lycopene, retinol, ascorbate, β-carotene, α-tocopherol, and γ-tocopherol) were selected from published studies. Data for alopecia areata (AA) and androgenetic alopecia (AGA) was obtained from the FinnGen study project (R9 released in 2023), including 195 cases and 201,019 controls for AGA and 682 cases and 361,140 controls for AA. We used the inverse variance weighted method as the primary MR method. Three additional methods were used as sensitivity analysis to validate the robustness of the results. We found a causal relationship between absolute β-carotene levels and AGA risk (*P* = .039), but not with AA (*P* = .283). The results of Wald ratio showed a protective effect of absolute β-carotene levels against AGA, with per 0.1 ln-transformed β-carotene being associated with a 76% lower risk of AGA (OR: 0.24, 95% CI: 0.06–0.93). Based on the fixed effects inverse variance weighting results, we found that α-tocopherol was protective against both AGA (*P* = .026) and AA (*P* = .018). For each unit increase in α-tocopherol, the effects of change in AGA and AA were 0.02 (95% CI: 0.00–0.61) and 0.10 (95% CI: 0.01–0.67), respectively. The results did not reveal any other causal relationships. Our study identified 3 causal associations of antioxidants with the risk of non-scarring alopecia. These results provide new insights into the prevention of non-scarring alopecia through diet.

## 1. Introduction

Androgenetic alopecia (AGA) and alopecia areata (AA) are the 2 most common types of non-scarring alopecia. Although AGA is known as male pattern baldness, it is also one of the common types of chronic hair loss in women. Incidence of AGA increases with age, affecting more than 80% of men and 50% of women over the age of 70.^[1,2]^ The AGA is characterized by changes in the hair growth cycle, including a shorter anagen phase time and a longer telogen phase time.^[2]^ Common symptoms of AGA in men include receding hairline and thinning hair on top of the head, but the hairline stays the same in women.^[3,4]^ AA is a common autoimmune skin disease characterized by an immune attack on hair follicles, resulting in patches of non-scarring alopecia, which affects approximately 2% of the global population.^[5,6]^ Many factors interact in the development of hair loss. In addition to conventional risk factors, such as complicated genetic susceptibility, advanced age, and lifestyle, evidence from previous studies suggests that oxidative stress plays a critical in the development of hair loss.^[7–9]^ Previous Mendelian randomization (MR) analysis found that shortened leukocyte telomere length was associated with the risk of non-scarring alopecia.^[10]^ Importantly, oxidative stress accelerates telomere attrition, leading to telomere shortening.^[11–13]^ Hence, new insights into the treatment of non-scarring alopecia may be gained by elucidating the causal role of antioxidants in the disease.

Oxidative stress, an imbalance between antioxidants and pro-oxidants, is thought to increase DNA damage and cause hair loss. Earlier research demonstrated that oxidative stress triggers the activation of the P53 pathway, inducing cell cycle arrest in the hair follicles and skin.^[14]^ In order to sustain intracellular balance, antioxidants must regulate pro-oxidant activity, employing 4 lines of defense.^[15–17]^ The primary defense against imbalance involves antioxidant enzymes, aimed at preventing free radical formation and neutralizing existing radicals. The succeeding defense comprises free radical scavengers like ascorbate, carotene, retinol, and others, countering free radicals via electron donation. The third and fourth defense lines aim to eradicate molecular and cellular damage induced by pro-oxidants. It is widely acknowledged that dietary antioxidants, including ascorbate (vitamin C), are the most easily accessible form of antioxidant protection. Adequate vitamins are vital for normal cell growth and function, and their insufficiency can cause hair loss. Despite the easy accessibility and affordability of diet-based supplements, it is crucial to identify the specific vitamins that mitigate the risk of hair loss.

It is considered a gold standard to establish causality through randomized controlled trials (RCTs).^[18]^ However, it is important to acknowledge that RCTs may not always be feasible due to their high expenses and ethical constraints. Numerous causal inquiries cannot be addressed through RCTs, such as understanding the prolonged impacts of addictive or potentially harmful substances. An alternative statistical approach to explore causality in exposure-outcome relationships involves the utilization of instrumental variables. Econometricians initially developed the instrumental variable approach around a century ago and subsequently implemented it in medical statistics.^[19,20]^ The objective of MR analysis is to explore causal connections among traits employing genetic variations as instrumental variables.^[21–23]^ The emergence of extensive biobanks has enabled genetic analysis of traits with moderate heritability (e.g., diet). A genome-wide association analysis (GWAS) for antioxidants provides a deeper exploration of the causal relationship between antioxidants and disease.^[24–27]^ The primary objective of this study was to investigate the causal connection between circulating antioxidants and the occurrence of non-scarring alopecia (AGA and AA).

## 2. Materials and methods

### 2.1. Study design

**Figure 1.**
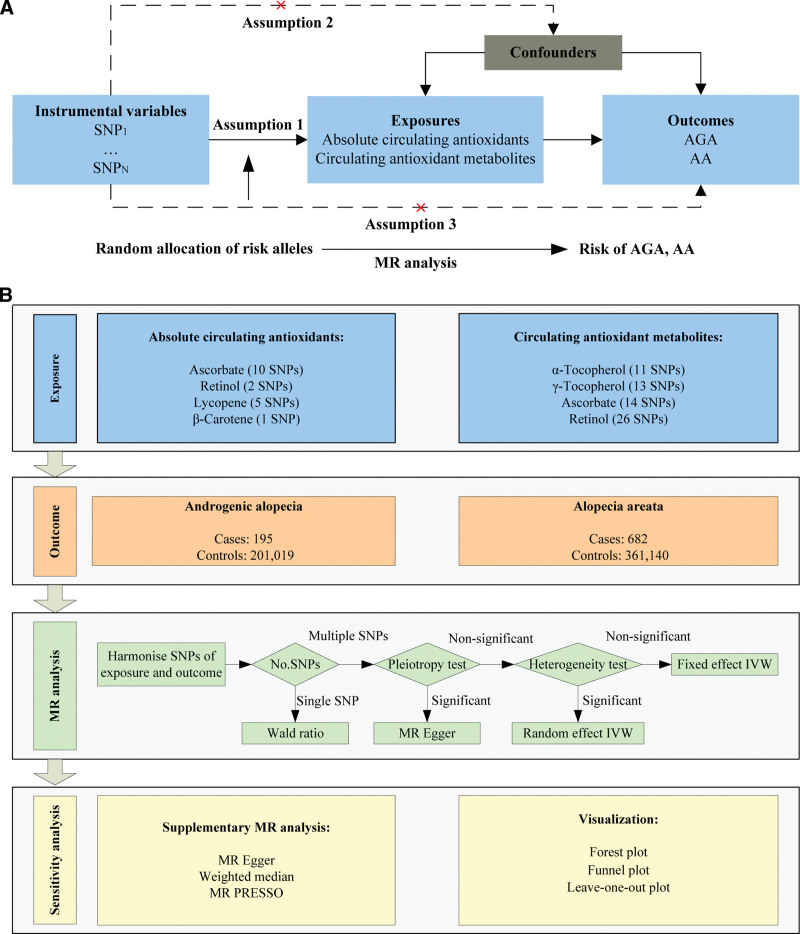
Schematic overview of the MR study design. (A) The 3 main assumptions of MR analysis. (B) Schematic overview and framework of the present MR study design. MR = Mendelian randomization.

The objective of this study was to investigate the causal links between diet-derived circulating antioxidants (ascorbate, lycopene, retinol, β-carotene, γ-tocopherol, and α-tocopherol) and non-scarring alopecia (AGA and AA) using 2-sample MR analysis based on GWAS summary data. Two phenotypes were selected for exposure data: absolute circulating antioxidants and circulating antioxidant metabolites. Genuine absolute blood levels were considered as absolute circulating antioxidants, whereas relative concentrations were considered as circulating antioxidant metabolites. MR analysis necessitates that all instrumental variables for circulating antioxidants meet 3 primary assumptions (Fig. 1A). Data on GWAS for AGA and AA were sourced from the FinnGen study. The research design framework is depicted in Figure 1B. Ethical approval was unnecessary as the study utilized publicly accessible data.

### 2.2. Genetic instrumental variables selection

The SNPs were identified as instrumental variables for ascorbate, retinol, and β-carotene from recent large-scale GWAS (*P* < 5 × 10 − 8, linkage disequilibrium (LD): r^2^ < 0.001, window size = 10,000 kb). In a recently published GWAS involving up to 52,018 individuals, 11 SNPs associated with ascorbate were found.^[28]^ Subsequently, one (rs7740812) of these SNPs was excluded after LD clumping. A GWAS meta-analysis involving 5006 participants across 2 cohort studies identified 2 independent SNPs related to retinol.^[29]^ A GWAS involving 2344 individuals from the Nurses’ Health Study detected 2 SNPs significantly associated with β-carotene.^[30]^ One (rs12934922) of the 2 SNPs was removed after LD clumping. In a GWAS involving 441 older Amish adults, 5 distinct SNPs related to lycopene were identified (*P* < 5 × 10 − 6, r^2^ < 0.001, window size = 10,000 kb).^[31]^ Supplementary Table 1, http://links.lww.com/MD/M740 contains cohort details for the GWAS employed in instrumental variable extraction.

For instrumental variables related to circulating antioxidant metabolites, we employed less stringent thresholds (*P* < 1 × 10 − 5, r^2^ < 0.001, window size = 10,000 kb). In brief, a GWAS involving 7824 adults from 2 European population studies provided independent SNPs linked to blood metabolites of α-tocopherol (n = 11), γ-tocopherol (n = 13), and ascorbate (n = 14).^[32]^ From 1960 participants of European ancestry, 26 SNPs were extracted as instrumental variables for retinol.^[33]^

Phenotype scanning, utilizing the PhenoScanner database, was conducted to explore the correlation of SNPs with confounding factors. SNPs linked to confounding factors were retained for further evaluation if they did not achieve genome-wide significance.^[34]^ The variance (R^2^) in the MR analysis is defined as the proportion of total variance explained by the genetic instrumental variables. To assess the strength of the instrumental variables, the *F*-statistic was also calculated.^[35]^

Details of the instrumental variables re shown in Table 1. The variance elucidated by the instrumental variables for circulating absolute antioxidants ranged from 1.7% to 30.1% (all F-statistics > 10), and for circulating antioxidant metabolites, the range spanned from 6.8% to 21.7% (all F-statistics > 10). Details of all SNPs utilized as instrumental variables are listed in Supplementary Tables 2, http://links.lww.com/MD/M741 and 3, http://links.lww.com/MD/M742.

**Table 1:** The summary of instrumental variables for diet-derived absolute circulating antioxidants and antioxidant metabolites.

Trait	Sample size	*P*	LD	No.SNPs	Explained variance (R^2^, %)	Unit	PMID
Absolute circulating antioxidants							
Ascorbate	52,018	5 × 10^−8^	0.001	10	1.7	µmol/L	33203707
Lycopene	441	5 × 10^−6^	0.001	5	30.1	µg/dL	26861389
Retinol	5006	5 × 10^−8^	0.001	2	2.3	µg/L in ln-transformed scale	21878437
β-Carotene	2344	5 × 10^−8^	0.001	1	4.8	µg/L in ln-transformed scale	23134893
Circulating antioxidant metabolites							
α-Tocopherol	7725	1 × 10^−5^	0.001	11	6.8	log10-transformed metabolites concentration	24816252
γ-Tocopherol	6226	1 × 10^−5^	0.001	13	9.8	log10-transformed metabolites concentration	24816252
Ascorbate	2085	1 × 10^−5^	0.001	14	21.7	log10-transformed metabolites concentration	24816252
Retinol	1960	1 × 10^−5^	0.001	26	20.6	log10-transformed metabolites concentration	28263315

LD: linkage disequilibrium. The R^2^ of each circulating antioxidant was extracted from the original study or calculated based on the following formula: R^2^ = (2 × EAF × (1-EAF) × Beta^2^)/[(2 × EAF × (1-EAF) × Beta^2^) + (2 × EAF × (1-EAF) × N × SE^2^)]. EAF is the effect allele frequency, Beta indicates the estimated genetic effect of SNP, N is sample size, and SE is standard error of the estimated effect.

### 2.3. Outcome data sources

The FinnGen study, as of its R9 release in 2023, represents an integration of genotype information and health registry data, encompassing a vast cohort exceeding 377,200 individuals, an extensive array of approximately 20.2 million genetic variants, and a comprehensive coverage of 2272 disease phenotypes. Within the FinnGen study, there were 195 cases identified along with 201,019 controls for AGA, and for AA, the study identified 682 cases and included 361,140 corresponding controls. Importantly, the study ensured there was no overlap in samples between the exposed cohort and the observed outcomes.

### 2.4. Statistical analysis

An overview of the primary MR analysis method selection process can be found in Figure 1B. Firstly, we harmonized the exposure and outcome information using effector allele of SNPs. For analysis with a single SNP, we used the Wald ratio as the primary method. In cases where multiple SNPs were available, the MR Egger intercept test was used to assess pleiotropy. MR Egger was used as the primary method for MR analysis when pleiotropy was present, otherwise Inverse Variance Weighting (IVW) was used. In the next step, Cochran Q test was used to assess heterogeneity. The random effects IVW model was used when heterogeneity was present, otherwise the fixed effects IVW model was used. Supplementary MR analysis, incorporating diverse assumptions, was conducted to confirm the causality established in the primary MR analysis. Using the MR Egger method, the study calculated causal effect values resistant to pleiotropy based on standard errors (SNPs > 2). A valid causal estimate can be presented by the weighted median if at least half of the weights account for valid instrumental variables (SNPs > 2). MR PRESSO was employed for outlier detection and subsequent correction. The threshold for significance was set at *P* < .05. The MR analysis in this study was carried out using the “TwoSampleMR (version 0.5.6)” package within the R software (version 4.2.2).

## 3. Results

### 3.1. Absolute circulating antioxidants and non-scarring alopecia

**Figure 2.**
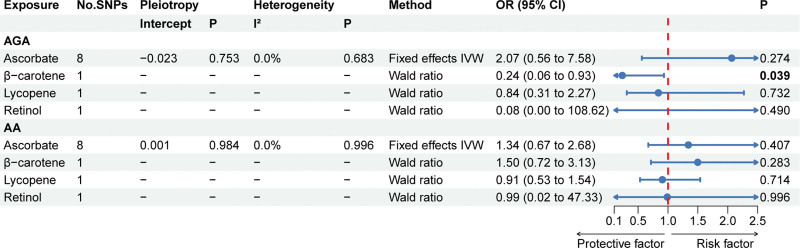
The primary MR analyses results of the causal effects of absolute circulating antioxidant levels on AGA and AA. Significant and suggestive results highlighted in bold. “-” represents not applicable. AA = alopecia areata, AGA = androgenetic alopecia, MR = Mendelian randomization.

The primary MR results concerning absolute antioxidants are illustrated in the forest plot of Figure 2. For ascorbate, the *P*-values of the MR Egger intercept test for AGA and AA were 0.863 and 0.996 respectively, indicating that there was no pleiotropy. Similarly, no evidence of heterogeneity was found using the Cochran Q statistic (*P* for AGA: 0.683, *P* for AA: 0.996). As β-carotene, lycopene, and retinol had only one SNP as instrumental variable, sensitivity analyses could not be performed. A causal relationship was discovered between β-carotene and decreased risk of AGA (*P* = .039), while no significant association was identified for AA (*P* = .283). Results of Wald ratio showed a protective effect of absolute β-carotene levels against AGA, with per 0.1 ln-transformed β-carotene being associated with a 76% lower risk of AGA (OR: 0.24, 95% CI: 0.06 to 0.93). As for the relationships of the other 3 diet-derived antioxidants (ascorbate, lycopene, and retinol) with AGA and AA, no significant evidence was found in the fixed effects IVW analysis or Wald ratio analysis (*P*: .274–.732).

**Figure 3.**
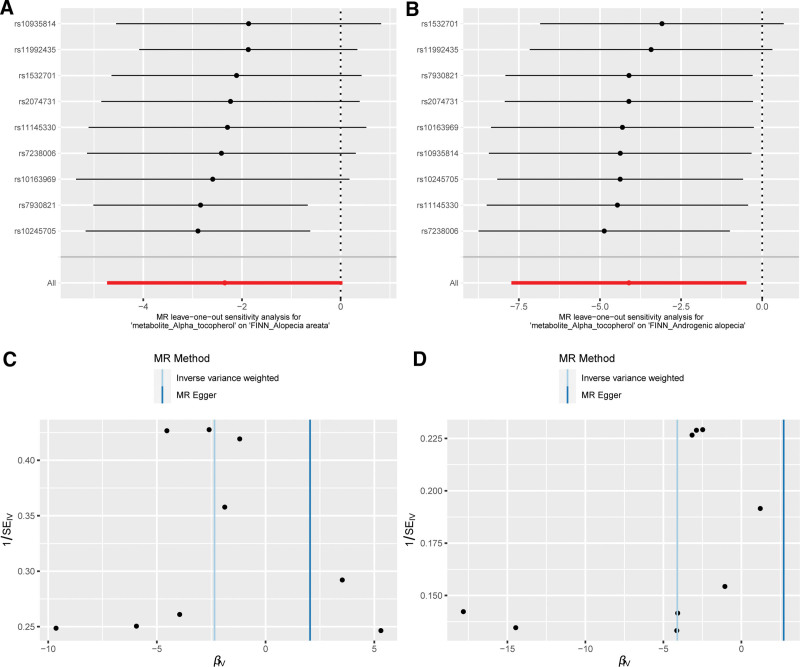
The leave-one-out plots and funnel plots for significant results. (A) Leave-one-out plot for α-tocopherol on AA. (B) Leave-one-out plot for α-tocopherol on AGA. (C) Funnel plot for α-tocopherol on AA. (D) Funnel plot for α-tocopherol on AGA. AA = alopecia areata, AGA = androgenetic alopecia.

The results of the complementary MR analysis methods were in excellent agreement with the IVW results, which did not show a cause-and-effect association between absolute ascorbate levels and AGA or AA (Supplementary Table 4, http://links.lww.com/MD/M743). The leave-one-out analysis did not identify a single SNP that caused a change in the direction of the MR results (Fig. 3A). Symmetrical distribution of the funnel plot generated by the IVW method (Fig. 3C). No outliers, which could lead to biased results, were found using the MR PRESSO method.

### 3.2. Circulating antioxidant metabolites and non-scarring alopecia

**Figure 4.**
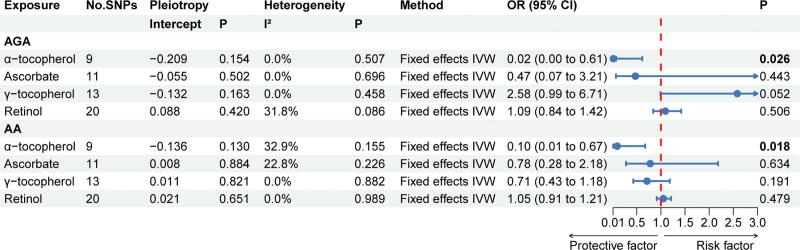
The primary MR analyses results of the causal effects of circulating antioxidant metabolites on AGA and AA. Significant and suggestive results highlighted in bold. “-” represents not applicable. AA = alopecia areata, AGA = androgenetic alopecia, MR = Mendelian randomization.

The primary MR results regarding antioxidant metabolites are depicted in the forest plot of Figure 4. The *P* value of the MR Egger intercept test ranged from 0.154 to 0.884, indicating that there is no pleiotropy. The *P* value of the Cochran Q test ranged from 0.086 to 0.989, indicating that there is no heterogeneity. Based on the fixed effects IVW results, we found that α-tocopherol was protective against both AGA (*P* = .026) and AA (*P* = .018). For each unit increase in α-tocopherol, the effects of change in AGA and AA were 0.02 (95% CI: 0.00–0.61) and 0.10 (95% CI: 0.01–0.67), respectively. As for the relationships of the other 3 diet-derived antioxidant metabolites (γ-tocopherol, ascorbate, and retinol) with AGA and AA, no significant evidence was found in the fixed effects IVW analysis (*P*: .052–.634).

The results of the complementary MR analysis methods for 3 diet-derived antioxidant metabolites (γ-tocopherol, ascorbate, and retinol) were in excellent agreement with the IVW results, which did not show a cause-and-effect association between absolute ascorbate levels and AGA or AA (Supplementary Table 5, http://links.lww.com/MD/M744). Although the results of complementary MR methods based on MR Egger and Weighted median did not show an association between α-tocopherol and the risk of AGA and AA, the fixed effects IVW was the most statistically valid method based on our screening of primary MR analysis methods. No outliers were found using the MR PRESSO method. The leave-one-out analysis did not identify a single SNP that caused a change in the direction of the MR results (Fig. 3B). Symmetrical distribution of the funnel plot generated by the IVW method (Fig. 3D).

## 4. Discussion

Due to the substantial rise in non-scarring alopecia incidence in recent decades, recognizing modifiable risk factors, particularly dietary elements, offers a promising strategy to curb the onset and advancement of these conditions. Using large GWAS data on circulating antioxidants and non-scarring alopecia, we found 3 causal associations, including absolute circulating antioxidant β-carotene with AGA (*P* = .039), circulating antioxidant metabolite α-tocopherol with AGA (*P* = .026), and circulating antioxidant metabolite α-tocopherol with AA (*P* = .018).

Some authors suggested that increased dietary intake of antioxidants could contribute to a reduction in the incidence of non-scarring alopecia, while other studies reported different results, indicating that higher levels of antioxidants were not associated with the risk of non-scarring alopecia. Clinical research has demonstrated an oxidant/antioxidant discrepancy in patients with AA.^[36]^ Vitamin E (α-tocopherol, γ-tocopherol) is involved in the balance between antioxidants and pro-oxidants, helping to prevent free radical damage. The results of a randomized case control study involving 15 AA patients and 15 healthy controls showed that vitamin E levels were significantly lower in AA patients than in healthy controls.^[37]^ However, a controlled study by Naziroglu and Kokcam, which included 37 patients with hair loss and 34 healthy controls, found no significant difference in plasma vitamin E levels between the 2 groups.^[38]^ In our MR study, we found that α-tocopherol was protective against both AGA (*P* = .026) and AA (*P* = .018). Vitamin C (ascorbate) is a natural nutrient with antioxidant properties, preventing free radical damage and oxidation of low-density lipoproteins. Although vitamin C deficiency is often associated with abnormal body hair,^[39]^ no study has linked vitamin C levels to hair loss. Our MR analysis showed that dietary vitamin C levels were not associated with the risk of AGA or AA. Vitamin A (retinol) is involved in the function of the immune system and is necessary for the growth and differentiation of cells. There is a case report of excessive intake or over-supplementation of vitamin A leading to hair loss.^[40]^ In addition, some studies have reported inconsistent results between lycopene, β-carotene and risk of hair loss.^[38,41–46]^ In summary, our MR analysis identified 3 causal relationships between diet-derived circulating oxidants and non-scarring alopecia including absolute β-carotene with AGA, metabolite α-tocopherol with AGA, and metabolite α-tocopherol with AA.

Our study demonstrates various strengths. Firstly, the MR design, employing 2 independent samples and instrumental variables, mitigates potential risks participants might face in clinical trials. Furthermore, using separate sets of instrumental variables for absolute circulating antioxidants and circulating antioxidant metabolites enhances the informativeness of the MR outcomes. Nevertheless, our study encounters limitations. Primarily, we acquired a restricted number of SNPs for antioxidant instrumental variables from published GWAS data. In other MR analysis, absolute lycopene, retinol and β-carotene were also unable to validate the robustness of the results using complementary MR analysis methods due to limitations in the number of SNPs.^[24,29,30]^ However, these SNPs reside within crucial genes associated with antioxidant metabolism and show no linkage to other non-scarring alopecia risk factors in the PhenoScanner database.^[34]^ Moving forward, it becomes imperative to identify more pertinent loci through expanded GWAS to augment the potency of the instrumental variables.

## 5. Conclusion

In conclusion, an increase in absolute β-carotene levels reduced the risk of AGA but was not associated with the risk of AA. In addition, levels of the circulating antioxidant metabolite α-tocopherol were negatively associated with both AGA and AA risk. More SNPs as proxies for circulating antioxidants are required for future MR analyses to extend the current findings based on large-scale GWAS.

## Author contributions

**Conceptualization:** Yuchen Ba, Junwen Wang.

**Data curation:** Lele Shen, Xiangning Peng, Yujin Zhang.

**Formal analysis:** Lele Shen, Xiangning Peng, Yujin Zhang.

**Funding acquisition:** Yuchen Ba, Junwen Wang.

**Methodology:** Lele Shen, Xiangning Peng, Yujin Zhang.

**Writing – original draft:** Yuchen Ba.

**Writing – review & editing:** Lele Shen, Junwen Wang.

## Supplementary Material










