# Supplementary material for: Elucidating causal relationships of diet-derived circulating antioxidants and the risk of non-scarring alopecia: A Mendelian randomization study
Source: Medicine (Baltimore). 2024 Jun 14;103(24):e38426. doi: 10.1097/MD.0000000000038426 (PMC11175974; doi:10.1097/MD.0000000000038426)
Supplement: Supplementary file 1 [file medi-103-e38426-s001.docx]

**Supplementary Table 1** The summary information for circulating antioxidants.

| Trait | Sample size | Age (years) | Sex (male, %) | Measurement method | Concentration |
| --- | --- | --- | --- | --- | --- |
| Absolute circulating antioxidants | | | | | |
| Ascorbate (umol/L) | | | | | |
| Fenland GWAS array | 1,349 | 45±7.0 | 44% | fluorometric assay | 66.2±21.3 |
| Fenland UKBB array | 8,391 | 49±7.0 | 47% | fluorometric assay | 68.6±21.5 |
| InterAct subcohort GWAS | 3,521 | 51±9.0 | 35% | high-performance liquid chromatography with ultraviolet detection | 42.8±19.0 |
| InterAct subcohort core-exome | 6,504 | 53±9.0 | 38% | high-performance liquid chromatography with ultraviolet detection | 42.9±19.1 |
| InterAct non-subcohort GWAS | 2,944 | 55±8.0 | 48% | high-performance liquid chromatography with ultraviolet detection | 36.4±17.6 |
| InterAct non-subcohort core-exome | 3,872 | 56±7.0 | 52% | high-performance liquid chromatography with ultraviolet detection | 36.5±18.9 |
| EPIC-Norfolk GWAS | 16,756 | 59±9.0 | 47% | fluorometric assay | 53.8±20.2 |
| EPIC-CVD subcohort | 885 | 53±12.0 | 41% | high-performance liquid chromatography with ultraviolet detection | 41.0±21.0 |
| EPIC-CVD non-subcohort | 6,765 | 57±8.0 | 55% | high-performance liquid chromatography with ultraviolet detection | 37.9±20.8 |
| Lycopene (µg/dL) | | | | | |
| HAPI | 441 | 43.1±13.0 | 58% | reverse-phase high-pressure liquid chromatography | 39.2±19.9 |
| Retinol (µg/dL) | | | | | |
| ATBC | 4,014 | 58.1±5.0 | 100% | reversed-phase liquid chromatography with diode-array UV detection | 572 (796-654) |
| PLCO | 992 | 64.6 ± 4.9 | 100% | reversed-phase liquid chromatography with diode-array UV detection | 672 (562-794) |
| β-Carotene (µg/L) | | | | | |
| NHS | 2,344 | 58.8±6.4 | 0% | reverse-phase high-pressure liquid chromatography | 303±258 |
| Circulating antioxidant metabolites | | | | | |
| α-tocopherol | | | | | |
| TwinsUK | 5,966 | 53.4±14.0 | 7% | liquid­phase chromatography and gas chromatography separation coupled with tandem mass spectrometry | NA |
| KORA | 1,759 | 60.8±8.8 | 49% |  | NA |
| γ-tocopherol | | | | | |
| TwinsUK | 5,249 | 53.4±14.0 | 7% | liquid­phase chromatography and gas chromatography separation coupled with tandem mass spectrometry | NA |
| KORA | 977 | 60.8±8.8 | 49% |  | NA |
| Ascorbate | | | | | |
| TwinsUK | 518 | 53.4±14.0 | 7% | liquid­phase chromatography and gas chromatography separation coupled with tandem mass spectrometry | NA |
| KORA | 1,567 | 60.8±8.8 | 49% |  | NA |
| Retinol | | | | | |
| TwinsUK | 1,960 | 58 (32–87) | NA | The non-targeted metabolomics analysis was performed at Metabolon (Durham, North Carolina, USA) on a platform consisting of four independent ultra-high-performance liquid chromatography–tandem mass spectrometry instruments | NA |

ATBC: Alpha-Tocopherol, Beta-Carotene Cancer Prevention Study; EPIC: European Prospective Investigation into Cancer and Nutrition; InCH: InCHIANTI Study; KORA: The Cooperative Health Research in the Region of Augsburg; NHS: Nurses’ Health Study; PLCO: Prostate, Lung, Colorectal, and Ovarian (PLCO) Cancer Screening Trial; HAPI: Heredity and Phenotype Intervention Heart Study; NA, not applicable.
