# Supplementary material for: Elucidating causal relationships of diet-derived circulating antioxidants and the risk of non-scarring alopecia: A Mendelian randomization study
Source: Medicine (Baltimore). 2024 Jun 14;103(24):e38426. doi: 10.1097/MD.0000000000038426 (PMC11175974; doi:10.1097/MD.0000000000038426)
Supplement: Supplementary file 2 [file medi-103-e38426-s002.docx]

**Supplementary Table 2** Instrumental variables associated with absolute circulating antioxidants.

| Antioxidant | SNP | Effect allele | Other allele | EAF | *F*-statistic^†^ | Beta | SE | *P* |
| --- | --- | --- | --- | --- | --- | --- | --- | --- |
| Ascorbate | rs6693447 | T | G | 0.55 | 42.25 | 0.039 | 0.006 | 6.25E-10 |
|  | rs13028225 | T | C | 0.86 | 128.44 | 0.102 | 0.009 | 2.38E-30 |
|  | rs33972313 | C | T | 0.97 | 400.00 | 0.360 | 0.018 | 4.61E-90 |
|  | rs10051765 | C | T | 0.34 | 31.04 | 0.039 | 0.007 | 3.64E-09 |
|  | rs174547 | C | T | 0.33 | 26.45 | 0.036 | 0.007 | 3.84E-08 |
|  | rs117885456 | A | G | 0.09 | 42.25 | 0.078 | 0.012 | 1.70E-11 |
|  | rs2559850 | A | G | 0.60 | 93.44 | 0.058 | 0.006 | 6.30E-20 |
|  | rs10136000 | A | G | 0.28 | 32.65 | 0.040 | 0.007 | 1.33E-08 |
|  | rs56738967 | C | G | 0.32 | 34.31 | 0.041 | 0.007 | 7.62E-10 |
|  | rs9895661 | T | C | 0.82 | 62.02 | 0.063 | 0.008 | 1.05E-14 |
| Retinol | rs10882272 | C | T | 0.35 | 56.25 | -0.030 | 0.004 | 6.51E-15 |
|  | rs1667255 | C | A | 0.31 | 56.25 | 0.030 | 0.004 | 6.35E-14 |
| Lycopene | rs7680948 | A | T | 0.20 | 40.11 | -0.190 | 0.030 | 4.97E-09 |
|  | rs4635297 | A | C | 0.08 | 27.04 | 0.260 | 0.050 | 6.46E-07 |
|  | rs341075 | A | G | 0.02 | 26.19 | -0.870 | 0.170 | 5.75E-07 |
|  | rs6108801 | C | T | 0.04 | 28.44 | -0.480 | 0.090 | 4.07E-07 |
|  | rs2232315 | A | G | 0.03 | 24.34 | 0.740 | 0.150 | 1.26E-06 |
| β-Carotene | rs6564851 | G | T | 0.36 | 98.67 | 0.149 | 0.015 | 1.60E-24 |

†The F-statistic for each SNP was calculated by the following formula: *F*-statistic = Beta^2^/SE^2^. EAF: effect allele frequency.
