# Supplementary material for: Elucidating causal relationships of diet-derived circulating antioxidants and the risk of non-scarring alopecia: A Mendelian randomization study
Source: Medicine (Baltimore). 2024 Jun 14;103(24):e38426. doi: 10.1097/MD.0000000000038426 (PMC11175974; doi:10.1097/MD.0000000000038426)
Supplement: Supplementary file 3 [file medi-103-e38426-s003.docx]

**Supplementary Table 3** Instrumental variables associated with circulating antioxidant metabolites.

| **Antioxidant** | **SNP** | **Effect allele** | **Other allele** | **EAF** | ***F*-statistic**^†^ | **Beta** | **SE** | ***P*** |
| --- | --- | --- | --- | --- | --- | --- | --- | --- |
| α-Tocopherol | rs10935814 | A | G | 0.10 | 19.87 | -0.037 | 0.008 | 9.44E-06 |
|  | rs1404410 | G | C | 0.21 | 20.60 | 0.024 | 0.005 | 4.57E-06 |
|  | rs10245705 | T | C | 0.02 | 27.25 | -0.066 | 0.013 | 1.95E-07 |
|  | rs11992435 | G | A | 0.05 | 20.63 | -0.033 | 0.007 | 6.38E-06 |
|  | rs11145330 | C | A | 0.11 | 22.70 | -0.032 | 0.007 | 1.95E-06 |
|  | rs7930821 | T | C | 0.02 | 19.98 | 0.067 | 0.015 | 7.53E-06 |
|  | rs261342 | C | G | 0.79 | 20.86 | -0.017 | 0.004 | 5.41E-06 |
|  | rs1532701 | A | G | 0.55 | 20.85 | 0.014 | 0.003 | 5.07E-06 |
|  | rs10163969 | T | G | 0.04 | 19.36 | -0.035 | 0.008 | 9.38E-06 |
|  | rs7238006 | C | T | 0.07 | 24.30 | -0.028 | 0.006 | 6.77E-07 |
|  | rs2074731 | A | C | 0.17 | 22.26 | -0.018 | 0.004 | 2.31E-06 |
| γ-Tocopherol | rs6713914 | C | T | 0.43 | 26.13 | -0.059 | 0.012 | 3.22E-07 |
|  | rs13069990 | T | C | 0.38 | 21.16 | -0.051 | 0.011 | 4.44E-06 |
|  | rs6834631 | G | T | 0.04 | 23.93 | -0.131 | 0.027 | 1.03E-06 |
|  | rs13103690 | G | T | 0.46 | 20.77 | 0.047 | 0.010 | 5.20E-06 |
|  | rs6826474 | T | C | 0.04 | 23.06 | -0.138 | 0.029 | 1.56E-06 |
|  | rs2070006 | C | T | 0.63 | 20.90 | -0.051 | 0.011 | 4.76E-06 |
|  | rs11167905 | C | T | 0.15 | 24.03 | -0.080 | 0.016 | 9.83E-07 |
|  | rs9419004 | C | G | 0.19 | 20.35 | -0.254 | 0.056 | 6.53E-06 |
|  | rs7112460 | T | C | 0.07 | 23.63 | 0.108 | 0.022 | 1.14E-06 |
|  | rs8057559 | T | C | 0.03 | 19.74 | 0.140 | 0.031 | 9.10E-06 |
|  | rs8105491 | T | G | 0.15 | 22.24 | -0.070 | 0.015 | 2.30E-06 |
|  | rs808686 | A | G | 0.61 | 21.83 | 0.060 | 0.013 | 3.01E-06 |
|  | rs9606290 | A | G | 0.24 | 20.40 | 0.159 | 0.035 | 6.32E-06 |
|  | rs577596 | A | G | 0.33 | 24.74 | -0.057 | 0.011 | 6.68E-07 |
| Ascorbate | rs2794327 | T | C | 0.67 | 19.69 | -0.036 | 0.008 | 8.78E-06 |
|  | rs6821770 | A | G | 0.14 | 19.52 | 0.038 | 0.009 | 8.92E-06 |
|  | rs10077932 | T | C | 0.14 | 21.35 | -0.040 | 0.009 | 4.08E-06 |
|  | rs10520845 | A | C | 0.02 | 20.78 | 0.191 | 0.042 | 5.27E-06 |
|  | rs7038957 | C | T | 0.17 | 21.43 | 0.029 | 0.006 | 3.86E-06 |
|  | rs10492212 | T | C | 0.16 | 19.53 | -0.027 | 0.006 | 8.66E-06 |
|  | rs10466757 | T | A | 0.84 | 19.66 | -0.063 | 0.014 | 9.56E-06 |
|  | rs7350776 | G | C | 0.30 | 21.12 | -0.024 | 0.005 | 3.86E-06 |
|  | rs261301 | C | T | 0.87 | 22.56 | -0.032 | 0.007 | 2.06E-06 |
|  | rs13336771 | A | C | 0.17 | 20.15 | 0.062 | 0.014 | 7.39E-06 |
|  | rs1013104 | T | C | 0.44 | 21.16 | -0.021 | 0.005 | 3.83E-06 |
|  | rs1060467 | G | A | 0.41 | 26.81 | -0.023 | 0.005 | 2.61E-07 |
|  | rs5994305 | G | A | 0.17 | 24.52 | -0.031 | 0.006 | 7.15E-07 |
| Retinol | rs10019071 | A | G | 0.02 | 16.65 | 0.657 | 0.161 | 3.64E-06 |
|  | rs112293959 | G | A | 0.03 | 10.89 | -0.429 | 0.130 | 5.70E-06 |
|  | rs114515641 | G | T | 0.03 | 10.30 | 0.414 | 0.129 | 7.12E-06 |
|  | rs1153379 | A | G | 0.93 | 15.14 | -0.323 | 0.083 | 6.10E-06 |
|  | rs1176744 | C | A | 0.32 | 21.16 | -0.207 | 0.045 | 3.50E-07 |
|  | rs118025446 | A | G | 0.03 | 17.49 | -0.481 | 0.115 | 9.84E-06 |
|  | rs12955464 | G | C | 0.14 | 14.72 | -0.234 | 0.061 | 3.71E-06 |
|  | rs139726207 | G | A | 0.04 | 11.31 | 0.370 | 0.110 | 4.46E-06 |
|  | rs149113848 | G | C | 0.01 | 13.23 | -0.964 | 0.265 | 3.47E-06 |
|  | rs149478645 | G | A | 0.02 | 12.75 | -0.507 | 0.142 | 1.30E-06 |
|  | rs17005512 | C | G | 0.17 | 13.20 | -0.218 | 0.060 | 2.77E-06 |
|  | rs1842947 | G | A | 0.52 | 19.94 | -0.192 | 0.043 | 8.34E-07 |
|  | rs2147337 | G | T | 0.66 | 12.89 | 0.158 | 0.044 | 9.01E-06 |
|  | rs2367816 | G | A | 0.77 | 19.99 | 0.228 | 0.051 | 9.46E-06 |
|  | rs2417325 | T | C | 0.93 | 15.34 | 0.329 | 0.084 | 1.29E-06 |
|  | rs3890033 | C | T | 0.38 | 11.76 | 0.144 | 0.042 | 8.56E-06 |
|  | rs3898702 | T | C | 0.20 | 16.15 | -0.217 | 0.054 | 3.02E-06 |
|  | rs4135385 | G | A | 0.24 | 18.72 | 0.212 | 0.049 | 9.80E-06 |
|  | rs58411567 | A | G | 0.22 | 16.00 | -0.208 | 0.052 | 3.02E-07 |
|  | rs6550239 | A | G | 0.74 | 14.54 | -0.183 | 0.048 | 4.40E-06 |
|  | rs75308833 | T | C | 0.02 | 11.29 | -0.494 | 0.147 | 3.51E-06 |
|  | rs7926028 | T | G | 0.45 | 10.33 | -0.135 | 0.042 | 2.75E-06 |
|  | rs945817 | A | G | 0.19 | 25.00 | -0.275 | 0.055 | 6.46E-07 |
|  | rs9586119 | C | T | 0.07 | 18.29 | 0.355 | 0.083 | 3.34E-06 |
|  | rs117468033 | T | C | 0.01 | 26.13 | -0.961 | 0.188 | 8.40E-06 |
|  | rs568632536 | T | C | 0.03 | 14.39 | 0.531 | 0.140 | 8.08E-06 |

†The F-statistic for each SNP was calculated by the following formula: *F*-statistic = Beta^2^/SE^2^. EAF: effect allele frequency.
