# Supplementary material for: Elucidating causal relationships of diet-derived circulating antioxidants and the risk of non-scarring alopecia: A Mendelian randomization study
Source: Medicine (Baltimore). 2024 Jun 14;103(24):e38426. doi: 10.1097/MD.0000000000038426 (PMC11175974; doi:10.1097/MD.0000000000038426)
Supplement: Supplementary file 4 [file medi-103-e38426-s004.docx]

**Supplementary Table 4** The complementary MR analyses results for absolute circulating antioxidants.

| **MR method** | **Ascorbate** | | |
| --- | --- | --- | --- |
|  | **No.SNPs*** | **OR (95% CI)** | ***P*** |
| **AGA** |  |  |  |
| MR Egger | 8 | 2.755 (0.322 to 23.583) | 0.391 |
| Weighted median | 8 | 3.477 (0.732 to 16.520) | 0.117 |
| MR PRESSO^†^ |  | NA | NA |
| **AA** |  |  |  |
| MR Egger | 8 | 1.328 (0.425 to 4.152) | 0.643 |
| Weighted median | 8 | 1.387 (0.617 to 3.118) | 0.429 |
| MR PRESSO^†^ |  | NA | NA |

Significant results highlighted in bold. †: No significant outliers. AGA: androgenetic alopecia; AA: alopecia areata; NA, not applicable.
