# Supplementary material for: Elucidating causal relationships of diet-derived circulating antioxidants and the risk of non-scarring alopecia: A Mendelian randomization study
Source: Medicine (Baltimore). 2024 Jun 14;103(24):e38426. doi: 10.1097/MD.0000000000038426 (PMC11175974; doi:10.1097/MD.0000000000038426)
Supplement: Supplementary file 5 [file medi-103-e38426-s005.docx]

**Supplementary Table 5** The complementary MR analyses results for circulating antioxidant metabolites.

| **MR methods** | **α-Tocopherol** | | | **γ-Tocopherol** | | | **Ascorbate** | | | **Retinol** | | |
| --- | --- | --- | --- | --- | --- | --- | --- | --- | --- | --- | --- | --- |
|  | **No.SNPs** | **OR (95% CI)** | ***P*** | **No.SNPs** | **OR (95% CI)** | ***P*** | **No.SNPs** | **OR (95% CI)** | ***P*** | **No.SNPs** | **OR (95% CI)** | ***P*** |
| **AGA** |  |  |  |  |  |  |  |  |  |  |  |  |
| MR Egger | 9 | 15.068 (0.002 to 136471.438) | 0.578 | 13 | 13.021 (1.269- 133.564) | 0.054 | 11 | 1.634 (0.031- 86.348) | 0.814 | 20 | 0.789 (0.341 to 1.825) | 0.586 |
| Weighted median | 9 | 0.055 (0.000 to 7.048) | 0.242 | 13 | 2.566 (0.640- 10.291) | 0.184 | 11 | 1.121 (0.090- 14.008) | 0.929 | 20 | 1.051 (0.710 to 1.557) | 0.804 |
| MR PRESSO^†^ |  | NA | NA |  | NA | NA |  | NA | NA |  | NA | NA |
| **AA** |  |  |  |  |  |  |  |  |  |  |  |  |
| MR Egger | 9 | 7.720(0.033-1806.268) | 0.487 | 13 | 0.623(0.180- 2.155) | 0.47 | 11 | 0.653(0.049- 8.711) | 0.755 | 20 | 0.972(0.675- 1.400) | 0.88 |
| Weighted median | 9 | 0.098(0.007- 1.440) | 0.090 | 13 | 0.600(0.291- 1.239) | 0.168 | 11 | 0.682(0.162- 2.878) | 0.602 | 20 | 0.985(0.817- 1.188) | 0.875 |
| MR PRESSO^†^ |  | NA | NA |  | NA | NA |  | NA | NA |  | NA | NA |

Significant results highlighted in bold. †: No significant outliers. AGA: androgenetic alopecia; AA: alopecia areata; NA, not applicable.
